# Effects of Swimming at Different Water Temperatures on Muscle and Adipose Tissue Adaptation in Diet-Induced Obese Mice

**DOI:** 10.7150/ijms.121250

**Published:** 2026-02-11

**Authors:** Tzu-Jung Chou, Chia-Wen Lu, Yi-Ju Hsu, Chi-Chang Huang, Kuo-Chin Huang

**Affiliations:** 1 Department of Family Medicine, National Taiwan University Hospital Hsin-Chu Branch, Hsinchu, Taiwan.; 2 Graduate Institute of Clinical Medicine, National Taiwan University College of Medicine, Taipei, Taiwan.; 3 Department of Family Medicine, National Taiwan University Hospital, Taipei, Taiwan.; 4 Department of Family Medicine, National Taiwan University College of Medicine, Taipei, Taiwan.; 5 Graduate Institute of Sports Science, National Taiwan Sport University, Taoyuan, Taiwan.

**Keywords:** Obesity, Swimming exercise, Water temperature, Muscle fiber transition, Metabolic adaptation

## Abstract

**Background:**

Obesity is a major global health concern, leading to increased risk of metabolic diseases and mortality. Swimming, as a low-impact exercise, may provide metabolic benefits. However, the influence of water temperature on the metabolic and physiological responses remains underexplored. This study investigated the effects of water temperatures on muscle and adipose tissue adaptations in high-fat diet-induced obese mice.

**Methods:**

Obese mice were subjected to swimming at different water temperatures: 15°C, 25°C, or 32°C. Changes in body and tissue weight, grip strength, exhaustive swimming performance, and key metabolic parameters were assessed. Epididymal fat pads (EFP) and gastrocnemius muscles were collected for histological analyses (muscle fiber composition and adipose tissue remodeling), gene expression (*Ucp1, Pgc-1α, Prdm16, Cidea*), and western blot analyses (SIRT1-PGC-1α-FNDC5 pathway).

**Results:**

Swimming at 25°C and 32°C significantly reduced body weight and EFP weight, improved metabolic profiles and grip strength, whereas cold-water swimming (15°C) enhanced endurance performance. Histological analysis revealed reduced adipocyte size in the 25°C group, accompanied by increased oxidative (Type I) fibers across all swimming groups. Elevated *Pgc-1α* expression in EFP was particularly prominent at 25°C, and FNDC5 in muscle was most pronounced at 15°C. These findings highlight distinct temperature-dependent metabolic and muscular adaptations during swimming in obese mice.

**Conclusion:**

Moderate (25°C) and warm (32°C) water temperatures are optimal for reducing obesity-related metabolic dysfunctions and enhancing muscle strength, while cold water (15°C) improves endurance through oxidative muscle adaptation.

## Introduction

Obesity is a global public health concern, contributing to numerous metabolic disorders such as type 2 diabetes, dyslipidemia, and cardiovascular diseases^1^, ^2^. Prolonged consumption of high-fat diets (HFDs) not only leads to obesity but also causes intramyocellular lipid accumulation, which may negatively affect muscle function^3^. Exercise is known to enhance insulin sensitivity, fat oxidation, and muscle metabolism^4^. Among various types of physical activity, aquatic exercise has gained attention due to its low-impact nature and high energy expenditure, making it suitable for individuals with obesity and limited mobility^5^.

Although swimming is recognized for its cardiometabolic benefits, the influence of water temperature on metabolic and muscular adaptations remains underexplored. Warmer water temperatures may induce vasodilation, thereby improving blood flow and enhancing nutrient delivery while facilitating the removal of metabolic by-products^6^. Conversely, cold water induces vasoconstriction, which may reduce inflammation and alleviate pain and muscle soreness^7^. However, the role of water temperature in muscle fiber composition and adipose tissue remodeling remains unclear. Swimming has been shown to trigger slow-to-fast transitions in certain muscle types, indicating a complex adaptive response^8^. While Type I (slow-twitch) muscle fibers are known for their elevated mitochondrial density, abundant capillary networks, and high fatigue resistance^9^, how temperature modulates these adaptions is unclear. Furthermore, research on whether cold-water swimming effectively induces browning of white adipose tissue is inconsistent^10^, ^11^.

Elucidating the interaction between swimming and water temperature is crucial for optimizing exercise interventions for obesity and related metabolic disorders. We hypothesize that water temperature differentially affects adipose tissue remodeling, skeletal muscle adaptation, and metabolic health in obese mice.

## Methods

### Experimental design and animal model

**Figure 1:**
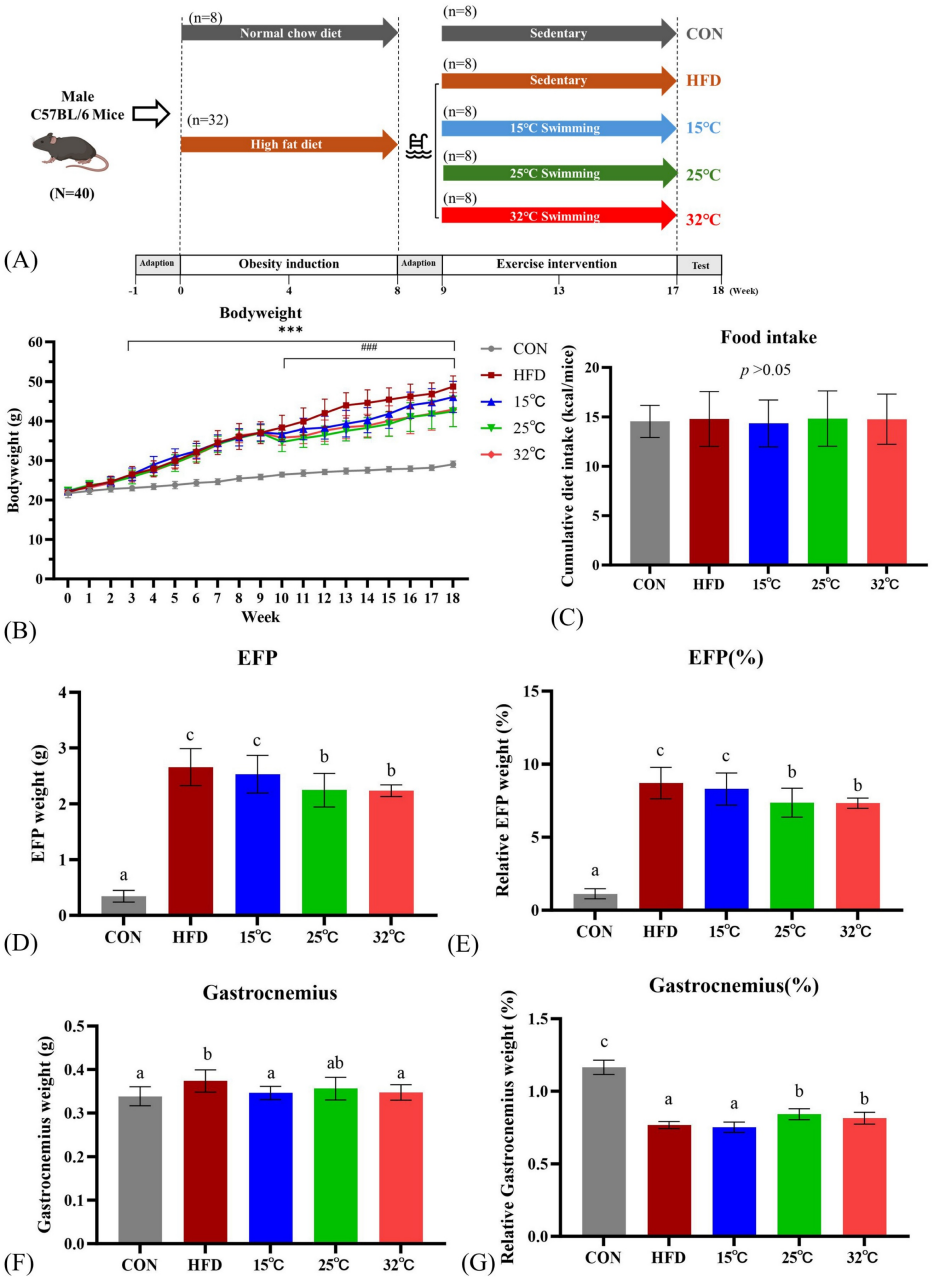
(A) Experimental design. (B) Body weight changes throughout the study period. (C) Cumulative caloric intake. (D, E) Absolute and relative epididymal fat pad (EFP) weights. (F, G) Absolute and relative gastrocnemius weights. Mice swimming at 25°C and 32°C showed significant reductions in body weight and EFP weight compared with HFD group. Different letters (a, b, c) indicate significant differences (*p* < 0.05) among groups based on *post hoc* comparisons. ***p < 0.001 versus CON group and ^###^ *p*< 0.001 versus HFD group. Data are presented as mean ± SD (n = 8 per group).

Forty male C57BL/6J mice aged five weeks were obtained from BioLASCO Taiwan. All animals were housed under standardized conditions: identical cage density, bedding, enrichment, and a 12 h/12 h light-dark cycle, controlled temperature (23-25 °C) and humidity (50%), with distilled water provided *ad libitum*. Cage positions were rotated to minimize location bias. Following one week of acclimation, mice were initially divided into the control group (CON) (n=8) and the high-fat diet (HFD) obesity induction group (n=32). The CON group received a standard chow diet (13.5% kcal from fat, 3.36 kcal/g, Laboratory rodent diet 5001; LabDiet), whereas the obesity induction group was fed HFD (60% kcal from fat, 5.24 kcal/g, Research Diets D12492; Research Diets, Inc.). All mice were pair-fed with isocaloric diets to minimize confounding effects from differences in food intake and to ensure that weight gain could be attributed primarily to the dietary fat composition rather than caloric intake^12^, ^13^. Previous studies have demonstrated that rodents fed a high-fat diet develop more pronounced obesity and insulin resistance than those maintained on isocaloric control diets^14^, ^15^. Obesity induction was considered established when the HFD group's average weight exceeded the CON group's by a minimum of 10%. Food intakes were recorded daily, and body weights were recorded bi-weekly. The experimental design is illustrated in Figure 1A. All procedures and protocols were approved by the Institutional Animal Care and Use Committee of the National Taiwan Sport University (IACUC-11001).

### Swimming exercise intervention

Mice were given one week to acclimate to swimming and determine the tolerable water temperature. The HFD group was subsequently randomly assigned into four groups using a random number generator: a sedentary HFD group (HFD) and three swimming training subgroups, categorized by water temperature - cold water swimming group at 15°C, mild below thermal neutral swimming group at 25°C, and the warm water swimming group at 32°C (n=8 per group). Sample size (n=8 per group) was determined using the Resource Equation approach^16^, ^17^. With 5 groups, the resulting error degrees of freedom (E = 35) exceed the minimum recommended range and still provide adequate statistical power. This sample size is consistent with established rodent exercise intervention studies^18^-^20^ and adheres to the 3Rs principle of minimizing animal use. The selection of water temperatures (15°C, 25°C, and 32°C) was based on physiological and metabolic considerations. Previous studies have established that 30-32°C represents the thermoneutral zone for mice, where metabolic stress is minimized^21^. A moderate water temperature of 25°C was chosen to mimic standard laboratory conditions and assess exercise-induced metabolic adaptations in a neutral thermal state^22^. The 15°C water temperature was included to investigate the impact of cold exposure on metabolic and muscle adaptations, as prior research suggests that cold-water swimming enhances endurance capacity and promotes oxidative muscle fiber transition^23^, ^24^.

The swimming exercise protocol was based on previous studies with slight modifications^25^, ^26^. Briefly, mice underwent 40 minutes of swimming exercise in a 45 × 45 × 45 cm water tank with a water depth of 15 cm five times weekly for eight weeks. Water temperature during swimming sessions was continuously monitored using a digital thermometer placed in the swimming tank, and maintained within ±0.5°C of the target temperature by adding ice or pre-warmed water as needed. Swimming sessions were closely monitored to ensure compliance and equal exposure, and mice were carefully towel-dried after each session. No running wheels or other exercise equipment were provided. Outside the intervention, staff performed daily visual checks (locomotion, grooming, posture) to identify any abnormal spontaneous activity.

### Exercise performance

Exercise performance was evaluated using forelimb grip strength and exhaustive swimming test. Forelimb grip strength was evaluated using a low-force testing system (Model-RX-5; Aikoh Engineering, Japan).^27^ The tensile force each mouse exerts was measured using a force transducer fitted with a 2 mm-diameter, 7.5 cm-long metal bar. Grip strength was measured 4 times per mouse with appropriate intervals to avoid fatigue. The highest force (in grams) recorded served as a measure of absolute grip strength, whereas relative grip strength was expressed relative to body mass. The test of forelimb grip strength was performed after obesity induction and 8 weeks after exercise intervention.

Endurance performance was assessed in a columnar pool maintained at 25 ± 1 °C. Swimming time was recorded from the start until exhaustion, defined as uncoordinated movements and the inability to surface within a 7-second timeframe.^19^ Mice swam freely without tail weights.

### Metabolic parameter measurements

Serum total cholesterol (TCHO), triglycerides (TG), low-density lipoprotein cholesterol (LDL), and high-density lipoprotein cholesterol (HDL) were determined by the Beckman DxC 800 autoanalyzer (Beckman Coulter, Brea, CA, USA) before and after 8 weeks of swimming intervention.

Oral glucose tolerance testing (OGTT) was performed as described previously^28^. Mice were fasted for 12 hours before testing. Tail vein glucose samples were obtained at baseline and 15, 30, 60, and 120 minutes following oral gavage of glucose (2 g/kg for all groups).

### Animal sacrifice and sample collection

Mice were euthanized using isoflurane inhalation and cervical dislocation after 12 hours of fasting. Trunk blood was collected and centrifuged (800 g, 20 min) to generate plasma. Epididymal fat pad (EFP) and gastrocnemius muscle tissue were extracted, weighed, immediately frozen in liquid nitrogen, and kept at -80 °C for subsequent analysis.

### Histology analysis

EFP and gastrocnemius muscle were fixed with 10% formalin, embedded in paraffin, and cut into 4 µm slices. These sections underwent Hematoxylin and eosin (H&E) staining for histological analysis. Muscle fiber types were distinguished by immunohistochemical analysis using myosin heavy chain fast (NCL-MHCf) and myosin heavy chain slow (NCL-MHCs) antibodies with the Bond-Max autostainer (Leica Biosystems)^29^. Briefly, this involved initial dewaxing of slides using Bond Dewax solution (Leica Biosystems) and rehydrating with Bond Wash solution (Leica Biosystems). Antigen retrieval was performed using Epitope Retrieval 2 solution (Leica Biosystems) at a pH of 9, heated to 100℃ for 20 minutes. Slides were then incubated with the primary antibody at a concentration of 1:100 for 60 minutes at room temperature. The detection kit used was the Bond Polymer Refine Detection (DS9800) and Bond Polymer Refine Red Detection (DS9390), incubated with post-primary for 8 minutes, polymer for 8 minutes, DAB for 5 minutes, and counterstained with Hematoxylin for 5 minutes. Images were captured by a light microscope (200x magnification) equipped with a CCD camera (BX-51, Olympus, Tokyo, Japan). Cross-sectional areas of the imaged adipocytes and muscle fibers were analyzed with Fiji Is Just ImageJ software supplemented by the Adiposoft and MuscleJ plugins.

### Quantitative real-time PCR

Total RNA was isolated from the frozen mice EFP via TOOLSmart RNA Extractor (DPT-BD24, Taiwan). RNA quality and concentration were assessed before further analysis. Quantitative real-time PCR analysis was performed using SYBR green one-step PCR Master Mix (Applied Biosystems, Carlsbad, CA). *Ucp-1* and other thermogenic genes (*Prdm16*, *Cidea*, and *Pgc-1α*) were evaluated in EFP as a hallmark of brown-like phenotype. All data were normalized to *Gapdh* mRNA content, and the primers are shown in Table S1.

### Western blotting

Gastrocnemius muscles were homogenized in lysis buffer (T-PER^TM^ Tissue Protein Extraction Reagent, Thermo Scientific) supplemented with protease and phosphatase inhibitors (ReadyShield Protease and Phosphatase Inhibitor Cocktail, Sigma-Aldrich). Protein quantities from each sample were evaluated utilizing the BCA protein assay kit (Thermo Scientific), followed by standardizing protein concentration before conducting all western blot experiments. Protein extracts from muscle lysates were separated into SDS-PAGE. Membranes were incubated with primary antibodies targeting SIRT1 (Cell signaling, catalog# 2496, 1:1000), PGC-1α (ABclonal, catalog# A12348, 1:1000), FNDC5 (Abcam catalog# ab174833, 1:1000), and β-actin (Cell signaling, catalog# 4970, 1:10000). The immunostaining was detected using horseradish peroxidase-conjugated anti-rabbit or anti-mouse IgG for 1 h at room temperature. For quantification, densitometry was performed using ImageJ, normalizing the target proteins against β-actin levels.

### Statistical analysis

Results were represented as mean ± standard deviation (SD) for normally distributed data and median ± interquartile range for skewed data. The normality of distribution for variables was assessed using the Shapiro-Wilk test. For normally distributed variables, comparisons among groups were performed using one-way analysis of variance (ANOVA) followed by Tukey's post hoc test to correct for multiple comparisons. For non-normally distributed variables, the Kruskal-Wallis test was used with Dunn's post hoc test and Bonferroni correction for multiple comparisons. To further evaluate changes within each group before and after the swimming intervention, paired t-tests were applied for normally distributed data and Wilcoxon matched-pairs tests for non-normally distributed data. Investigators performing histological assessments and molecular analyses were blinded to group allocation to minimize potential bias. Statistical analyses were performed using SPSS v22 (IBM, Armonk, New York) and GraphPad v9 (GraphPad Software, San Diego, CA). A *p*-value of less than 0.05 was considered statistically significant.

## Results

### Swimming at moderate and warm temperatures (25 °C and 32 °C) lowers body weight and fat pad weights

Body weight changes over time for each group are presented in Figure 1B. At baseline, body weights did not differ significantly between groups. After 8 weeks of obesity induction, the body weight of the HFD group was significantly higher than that of the CON group (*p* < 0.001). One week after the initiation of the swimming exercise, the body weights of the mice in the 25 °C and 32 °C groups showed a significant decrease compared to the HFD group (*p* < 0.001). During the training period, the 15 °C group showed a reduction in body weight, but the difference was not statistically significant. At the end of the study, swimming at 25 °C and 32 °C exhibited significant weight loss compared with the HFD group, while swimming at 15 °C showed no significant difference between the HFD group (15 °C 46.1±3.92 g, 25 °C 42.48±3.88 g, 32 °C 42.93±4.30 g, HFD 48.75±2.64 g, *p* < 0.05). Additionally, there was no significant difference among all groups in cumulative caloric intake (Fig. 1C).

Absolute and relative weights of EFP and gastrocnemius muscles are shown in Figure 1D-G. The HFD group revealed significantly higher absolute and relative EFP weights compared to the CON group. Swimming at 25 °C and 32 °C significantly reduced both absolute and relative EFP weights compared with the HFD group (Figure 1D, E). The absolute gastrocnemius weights were significantly higher in the HFD group compared to the CON group, suggestive of muscle fat infiltration (Figure 1F). Nevertheless, the 25 °C and 32 °C groups exhibited significantly higher relative gastrocnemius weights than the HFD and 15 °C groups (Figure 1G).

### Swimming in moderate and warm temperatures (25 °C and 32 °C) conditions enhances grip strength, while cold water (15 °C) increases endurance

**Figure 2:**
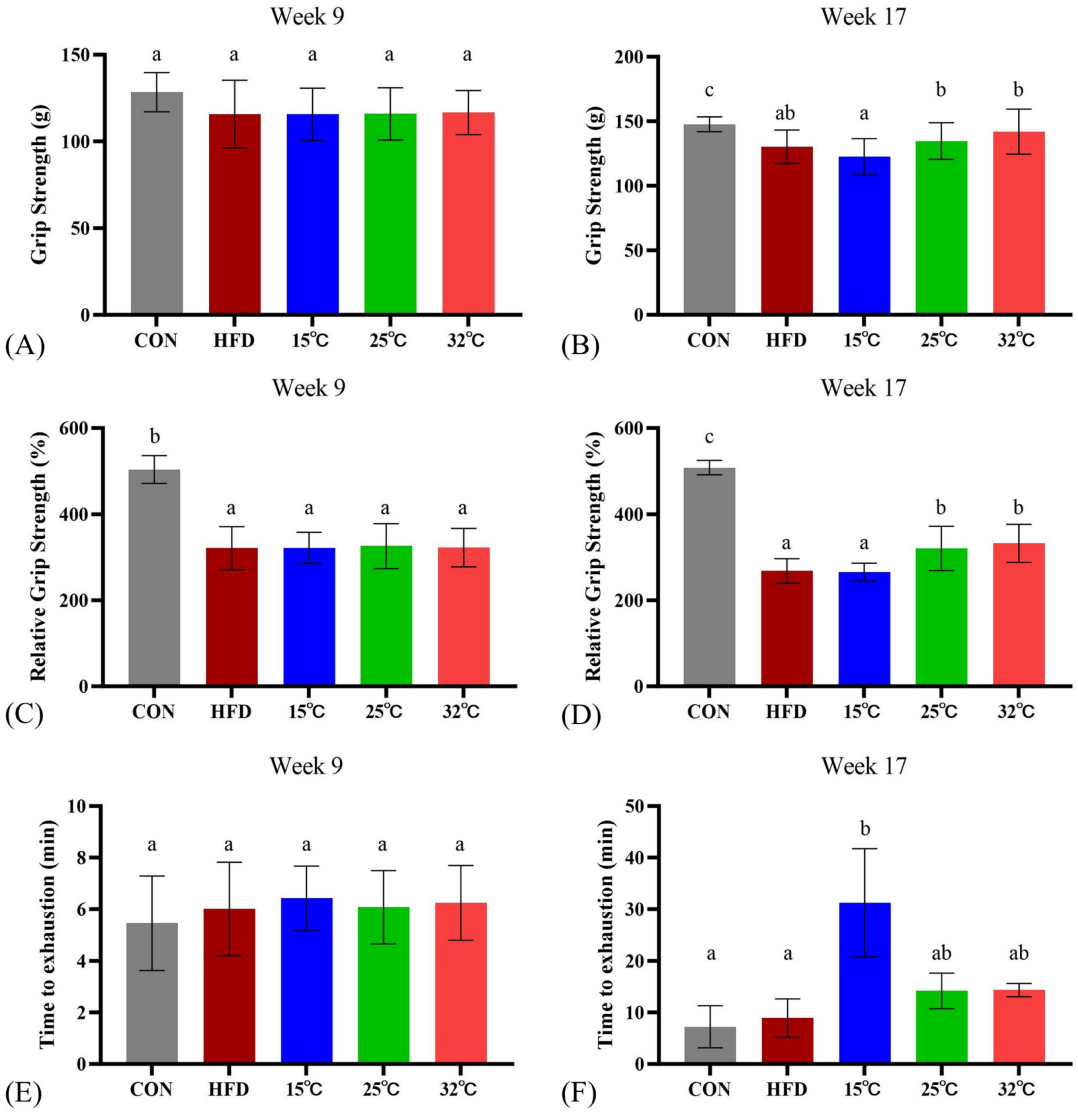
**Effects of swimming at different water temperatures on exercise performance.** Absolute grip strength (A, B), relative grip strength (C, D), and exhaustive swimming time (E, F) measured at Week 9 and Week 17 (after 8-week intervention). Grip strength was significantly improved in the 25°C and 32°C groups, while endurance capacity was enhanced in the 15°C group. Different letters (a, b, c) indicate significant differences among groups (*p* < 0.05) based on *post hoc* comparisons. Data are presented as mean ± SD (n = 8 per group).

Prior to the swimming intervention, the CON group significantly outperformed the HFD group in both absolute and relative grip strength (n = 8 per group, Figure 2AC). After 8 weeks of intervention, both 25 °C and 32 °C groups showed significantly enhanced relative grip strength compared with the HFD group and 15 °C groups, suggesting potential benefits of swimming in warmer water temperatures on grip strength (Figure 2BD). Interestingly, in the endurance test, measured by time until swimming exhaustion, the 15 °C group exhibited significantly longer endurance (Figure 2F). Consistently, within-group pre-post analyses (Supplementary Table S2) revealed significant increases in grip strength and relative grip strength in the 25 °C and 32 °C groups, and a marked prolongation of swimming time in the 15 °C group.

### Swimming at different temperatures differentially affects metabolic parameters in mice

**Figure 3:**
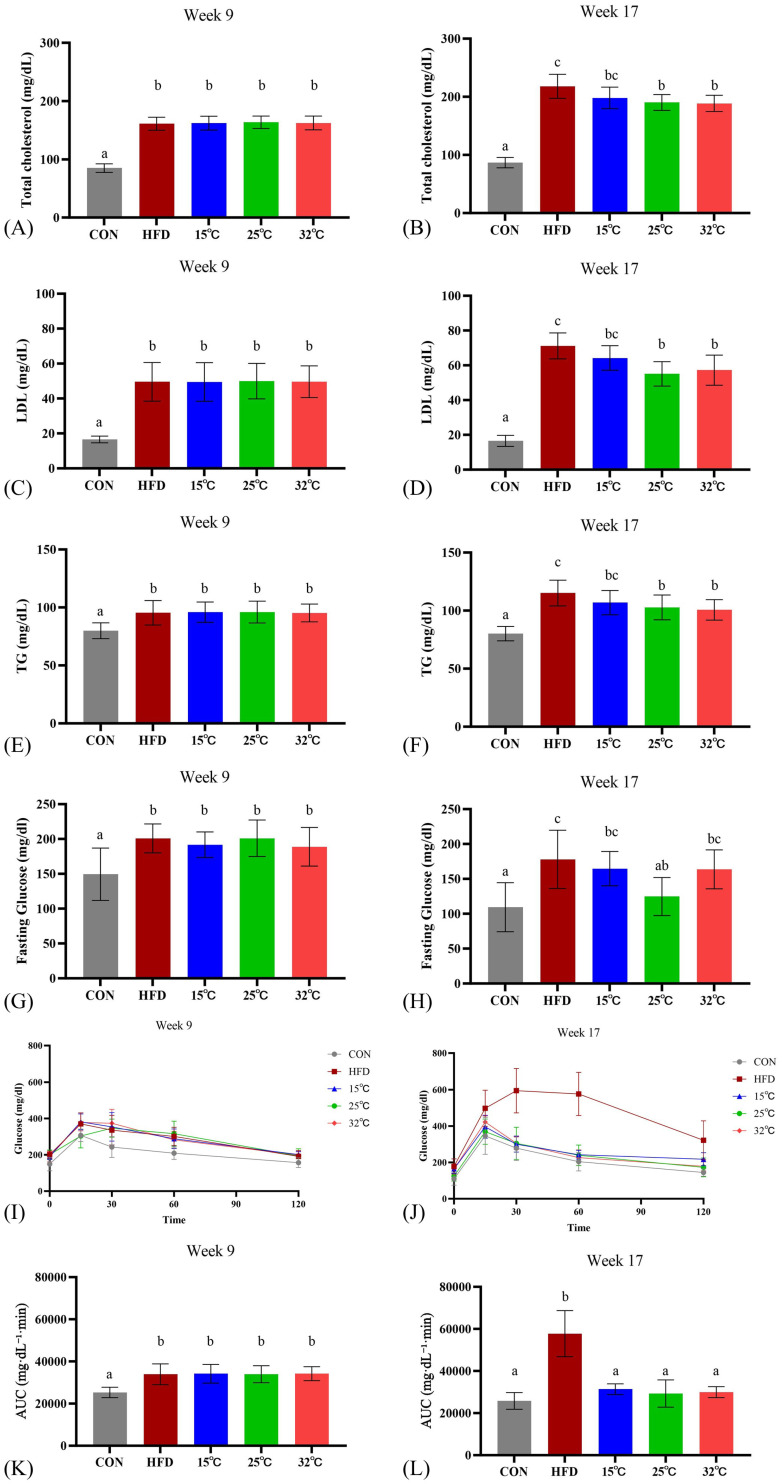
**Effects of swimming at different water temperatures on metabolic parameters.** (A, B) TCHO, (C, D) LDL, (E, F) TG, (G, H) fasting glucose, and (I, J) OGTT with corresponding (K, L) AUC at Week 9 and Week 17 (after 8-week intervention). Swimming at 25°C and 32°C significantly reduced lipid profiles, while only the 25°C group improved fasting glucose. All swimming groups showed improved glucose tolerance compared with HFD group. Different letters (a, b, c) indicate significant differences among groups (*p* < 0.05) based on *post hoc* comparisons. Data are presented as mean ± SD (n = 8 per group). TCHO = Total Cholesterol; LDL = Low-Density Lipoprotein Cholesterol; TG = Triglycerides; OGTT = Oral Glucose Tolerance Test; AUC = Area Under the Curve.

Before exercise intervention, the serum TCHO, LDL, TG, fasting glucose, and OGTT of the HFD groups were significantly increased compared with the CON group (n = 8 per group, *p* < 0.05; Figure 3 A, C, E, G, K). The effect of swimming at varying water temperatures produced mixed results after 8 weeks. Both the 25 °C and 32 °C groups showed significantly lower TCHO, LDL, and TG levels compared to the HFD group (*p* < 0.05), whereas the 15 °C group did not (Figure 3 B, D, F). HDL levels were also assessed, but did not show the typical exercise-induced elevation, possibly due to the continued high-fat diet consumption. Notably, only the 25 °C group showed improved fasting glucose levels (Figure 3H). All swimming temperature conditions resulted in a significantly improved glucose tolerance than the HFD group at 8 weeks (Figure 3 J, L). These findings suggested that the water temperature during swimming exerts differential effects on metabolic parameters, with moderate and warm temperatures demonstrating more pronounced improvements. Complementary within-group analyses (Supplementary Table S2) showed no significant pre-post changes in serum lipid or glucose parameters within individual groups, suggesting that the observed improvements were primarily temperature-dependent intergroup effects.

### Swimming at warmer temperature (25 °C and 32 °C) reduces EFP adipocyte size and improves muscle fiber composition

**Figure 4:**
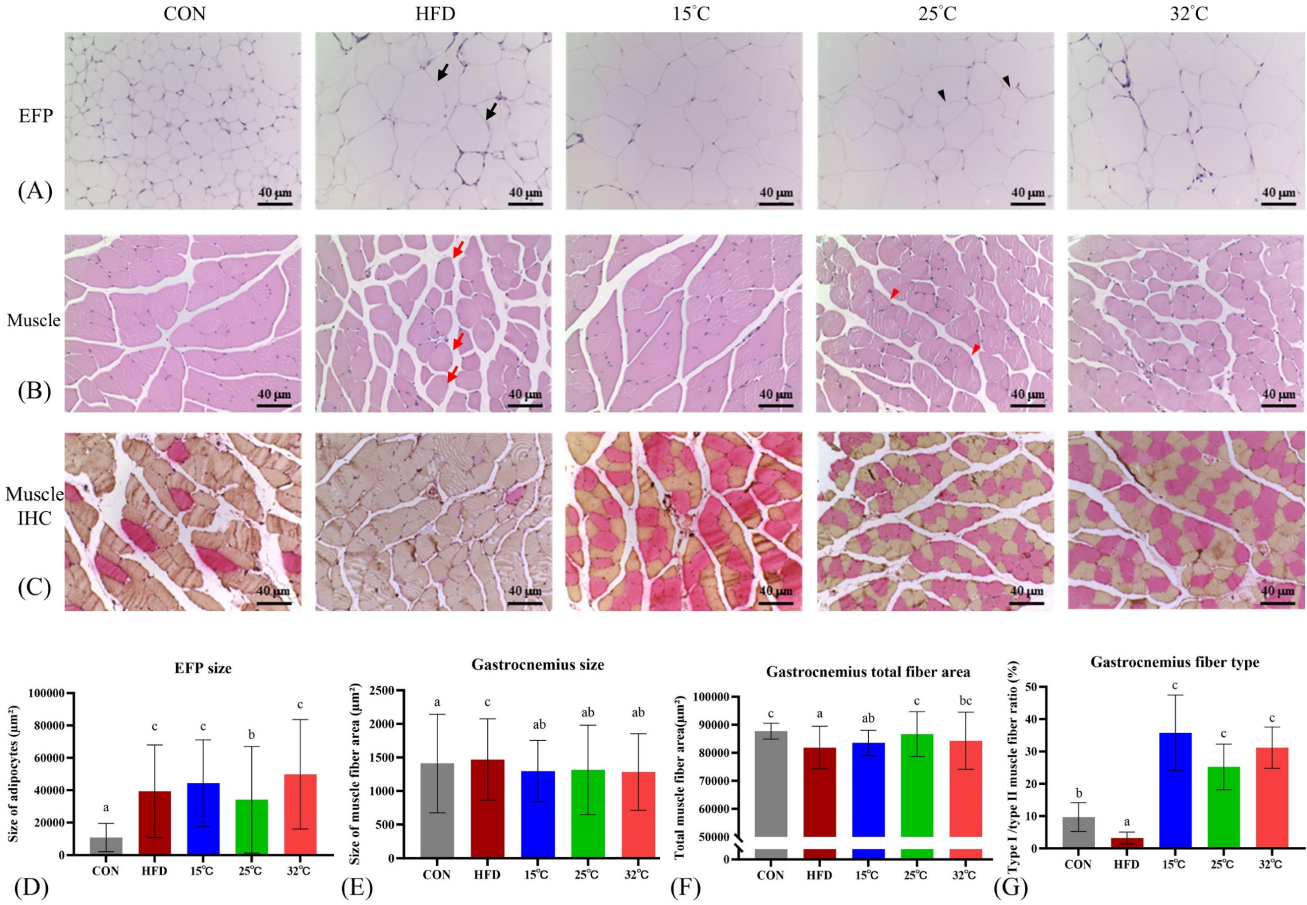
** Effects of swimming at different temperatures on EFP and gastrocnemius muscle.** (A) H&E staining of EFP. Black arrows indicate enlarged adipocytes in the HFD group; black arrowheads show smaller adipocytes in the 25 °C group. (B) H&E staining of gastrocnemius muscle. Red arrows show hypertrophic fibers with lipid infiltration in the HFD group; red arrowheads indicate restored, uniform fiber morphology in the 25 °C group. (C) Immunohistochemical staining of gastrocnemius showing Type I (red) and Type II (orange) fibers. (D-G) Quantitative analyses of EFP adipocyte size, gastrocnemius muscle fiber CSA, total fiber area, and Type I/II fiber ratio. The 25°C group showed significantly smaller adipocyte size and increased total muscle fiber area, while the 15°C group had the highest proportion of Type I fibers. Different letters indicate significant differences among groups (*p* < 0.05) based on *post hoc* comparisons. Data are presented as mean ± SD (n = 4 per group). Scale bar = 40 μm; magnification = 200×. CSA = Cross Sectional Area, EFP = Epididymal Fat Pads, H&E = Hematoxylin and Eosin.

Histological examination of EFP indicated marked adipocyte hypertrophy in HFD mice. The 25 °C group showed significantly smaller adipocyte size compared with HFD and the other swimming groups (Figure 4A, D). Additionally, the gastrocnemius muscle in mice is predominantly a fast-twitch (Type II) muscle with a relatively small slow-twitch (Type I) component^30^, and adaptations to HFD were muscle-specific^31^. Gastrocnemius muscle sections from HFD mice displayed increased fiber cross-sectional area (CSA), lower total fiber area, and a reduced proportion of Type I fibers (Figure 4B-G). Swimming intervention countered these alterations, with the 25°C group showing a notable increase in total fiber area, while all swimming groups exhibited increased Type I fiber proportions. Notably, the largest rise in Type I fibers was observed in 15 °C, which may account for this group's superior endurance capacity.

### Expression of thermogenic genes in EFP following different swimming temperatures

**Figure 5:**
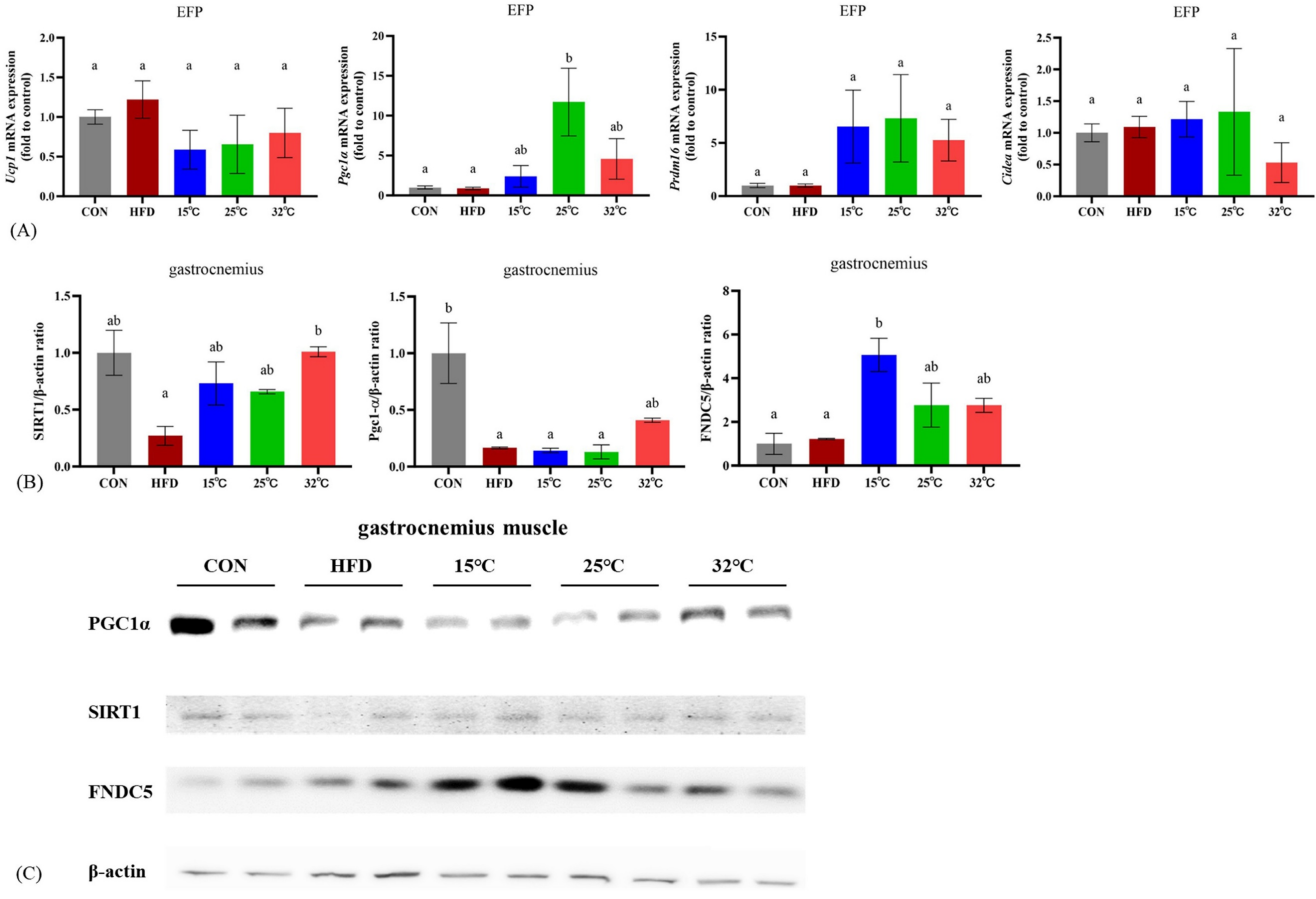
**Gene and protein expression in adipose tissue and skeletal muscle at different swimming temperatures.** (A) mRNA expression of *Ucp1*, *Pgc1α*, *Prdm16*, and *Cidea* in EFP (n = 4 per group). (B) Quantification of SIRT1, PGC-1α, and FNDC5 protein levels in gastrocnemius (n = 2 per group). (C) Representative immunoblots. *Pgc1α* was significantly upregulated in the 25°C group, whereas FNDC5 expression was highest in the 15°C group. Different letters (a, b) indicate significant differences among groups (*p* < 0.05) based on *post hoc* comparisons. Data are presented as mean ± SD. EFP = Epididymal Fat Pads.

To explore the effects of swimming at different temperatures on adipose tissue metabolism, we measured the expression levels of thermogenic genes in EFP (n = 4 per group) (Figure 5A). The 25 °C group showed significantly increased expression of* Pgc-1α* compared with CON and HFD groups (*p* < 0.05). There were no significant differences in *Ucp1*, *Prdm16*, and *Cidea* expression among groups.

### Effects of different swimming temperatures on the SIRT1-PGC-1α-FNDC5 pathway in gastrocnemius muscle

Western blot analyses of the gastrocnemius muscle revealed differential expression of key metabolic and mitochondrial regulators under different temperature conditions (Figure 5BC). All exercised groups showed higher levels of SIRT1 expression relative to the HFD group; however, only the 32 °C group showed a statistically significant increase. PGC-1α expression was significantly lower in the HFD group compared to the CON group (*p* < 0.05), while the 32 °C group exhibited a trend toward increased expression but not statistical significance. FNDC5 expression was significantly elevated in the 15 °C group compared to both the CON and HFD groups (*p* < 0.05). The 25 °C and 32 °C groups also exhibited higher FNDC5 expression, although these increases were not statistically significant. These findings suggest that although exercise at any temperature confers some metabolic benefits, each temperature elicits a unique regulatory profile for the SIRT1-PGC-1α-FNDC5 pathway.

## Discussion

Our study investigated how varying water temperatures (15 °C, 25 °C, and 32 °C) during swimming exercise affect metabolic health, exercise performance, adipose tissue characteristics, and muscle adaptations in diet-induced obese mice. The results demonstrated that all swimming groups induced muscle fiber transition, but moderate (25 °C) and warm (32 °C) swimming significantly reduced body weight and fat mass, and improved metabolic parameters, whereas cold-water swimming (15 °C) primarily improved endurance. These preclinical findings may have translational relevance. Water temperature could be an important factor when designing aquatic exercise interventions for obesity.

Muscle adaptations varied by water temperature, which may in turn affect exercise performance^6^, ^32^. D'Amico et al. found that increased expression of heat shock proteins (HSPs), such as Hsp60 and αB-crystallin, during warm water exercise supports muscle remodeling and adaptation by enhancing cellular stress responses and promoting protein stability, which may contribute to improved muscle function in moderate and warm temperatures^33^. In contrast, cold-water swimming (15 °C) may trigger stress responses—potentially mediated by elevated circulating glucocorticoids —that counteract weight loss by stimulating gluconeogenesis and fat storage^34^. Nevertheless, a cold environment in 15 °C water may slow the accumulation of muscle fatigue by reducing lactate build-up, allowing for longer swimming periods before exhaustion^35^. Bruton et al. found that cold-acclimated mice exhibit adaptations similar to endurance training, including increased calcium levels and mitochondrial content, which enhance fatigue resistance^24^. Additionally, muscle fiber transition may also affect exercise performance. Previous study has shown that diet-induced obesity can alter muscle fiber type, with type I fibers being most susceptible, especially in male mice^36^. Insulin resistance and metabolic dysfunctions in diet-induced obesity can be caused by alterations in muscle metabolism and muscle fiber phenotypes^37^. After swimming intervention, we found that the 15°C group had the greatest increase in Type I fibers, which may account for this group's superior endurance capacity. The 25 °C and 32 °C groups also exhibited an increase in Type I fibers compared to the HFD group; however, Type II fibers remained predominant, which likely explains the greater grip strength observed in these groups. Exercise can induce glycolytic muscle fibers to transition towards a more oxidative phenotype, which can upregulate myoglobin synthesis, enhance lipid utilization, and improve mitochondrial biogenesis and angiogenesis^38^.

Despite these differences, all groups exhibited improved insulin sensitivity, as reflected by OGTT results, highlighting the general metabolic benefits of swimming exercise. In addition to improvements in glucose tolerance and lipid metabolism, we also measured serum HDL levels, which showed no significant differences among the HFD and swimming groups. It is plausible that the continued consumption of high-fat diets throughout the intervention period may have attenuated the exercise-induced HDL response^39^. Previous studies have reported that high-fat feeding can blunt exercise-induced increases in HDL or alter HDL subclass distribution rather than total concentration^40^, ^41^. Moreover, improvements in HDL function—such as enhanced antioxidant capacity or cholesterol efflux—may occur without a detectable rise in plasma HDL levels^42^, ^43^.

The metabolic benefits of swimming may be partially mediated by the SIRT1-PGC-1α axis in skeletal muscle^44^, ^45^. SIRT1 enhances fatty acid oxidation and mitochondrial biogenesis^46^, while PGC-1α regulates oxidative capacity and glucose homeostasis^47^, ^48^. Increased *Pgc1-α* expression is known to promote oxidative muscle fiber type-switching, which could be more resistant to the atrophic effects of HFD^49^. It is plausible that the preserved muscle mass and increased grip strength in the 32°C group might be related to the enhanced mitochondrial activity and energy metabolism through the upregulation of *Pgc1-α*. However, FNDC5 expression did not increase as much in the 25°C and 32°C groups, indicating potential temperature-specific signaling pathways.

FNDC5, which is cleaved into irisin and regulates muscle-adipose communication^50^, was significantly elevated in the 15°C group. Cold exposure has been shown to alter gene expression in skeletal muscle, enhancing thermogenic pathways and muscle contractions^51^. One possible explanation is that cold-water swimming may induce additional muscle activity for thermogenesis, which could contribute to the increased FNDC5 expression. Additionally, Zhou et al. found that FNDC5's protective role during cold-induced stress facilitates muscle adaptation^52^. However, as irisin signaling involves multiple interacting pathways, our interpretation remains speculative and requires further investigation.

In EFP, swimming at 25°C induces favorable metabolic adaptations, including reduced adipocyte size and upregulation of metabolic genes such as *Pgc1α*, *Prdm16*, and *Cidea*—albeit some changes were not statistically significant. This trend suggests a partial shift toward a more oxidative and metabolically active adipose tissue phenotype (possibly “beige” adipocytes), though it did not involve robust elevations in Ucp1. Rahmani et al found that cold-water swimming promotes adipose tissue browning in Wistar rats by inhibiting Myostatin and increasing expression of IRF4, PGC-1α, and UCP1, enhancing thermogenesis and energy expenditure^53^. However, their study included weighted swimming (3-6% body weight), which likely imposed additional mechanical and metabolic stress, potentially amplifying UCP1 expression. The absence of a strong Ucp1 response in our study suggests that temperature alone may be insufficient to induce classical adipose browning without additional stimuli.

Our study had several limitations. First, the muscle fiber analysis did not differentiate subtypes of Type II fibers, limiting a more nuanced understanding of specific fiber adaptations. Second, as we did not measure circulating stress hormones, such as cortisol levels, it remains unclear whether different water temperatures induced varying levels of stress in mice. Further research is needed to determine the impact of thermal stress on metabolic and muscular adaptations. Third, although additional signaling pathways such as AMPK, Akt, and mTOR are known to mediate exercise-induced metabolic adaptations, these were not systematically investigated in the present study. Future research integrating these pathways would provide a more comprehensive understanding of the temperature-specific molecular responses to swimming exercise. Moreover, our study examined only three discrete water temperatures. Including a broader range of temperatures or finer increments could provide additional insights into the relationship between temperature and exercise-induced metabolic adaptations. However, our temperature selection was based on tolerability in mice, as extreme cold (< 15 °C) or warm (> 32 °C) conditions may cause excessive stress or hypothermia. Lastly, we observed relatively large variability in some datasets, which may reflect biological heterogeneity among diet-induced obese mice as well as methodological factors such as inter-individual differences in swimming tolerance or sample processing.

In conclusion, our study highlights distinct temperature-dependent outcomes of swimming exercise in diet-induced obese mice. Swimming at 25 °C and 32 °C effectively reduces body weight, enhances adipose tissue remodeling, and improves grip strength, whereas swimming at 15 °C primarily enhances endurance by promoting oxidative muscle fiber transition. These findings provide mechanistic insights and potential translational relevance for human aquatic exercise programs. Although direct extrapolation from mice to humans should be made cautiously, our results suggest that moderate water temperatures may optimize weight loss and metabolic outcomes, while cooler water may benefit endurance training. Future studies should further clarify the molecular pathways involved and determine optimal water temperatures for different exercise goals in obesity management.

## Supplementary Material

Supplementary tables.
