# Supplementary material for: Effects of Swimming at Different Water Temperatures on Muscle and Adipose Tissue Adaptation in Diet-Induced Obese Mice
Source: Int J Med Sci. 2026 Feb 11;23(3):1046–57. doi: 10.7150/ijms.121250 (PMC12964579; doi:10.7150/ijms.121250)
Supplement: Supplementary file 1 — Supplementary tables. [file ijmsv23p1046s1.pdf]

Supplementary Table S1. Forward and reverse primer sequences

Genes	Forward	Reverse
Ucp1	5’-CATGGGATCAAACCCCGCTA-3’	5’-ATTAGGGGTCGTCCCTTTCC-3’
Prdm16	5’-TGACCATACCCGGAGGCATA-3’	5’-CTGACGAGGGTCCTGTGATG-3’
Pgc1α	5’-CTGCGGGATGATGGAGACAG-3’	5’-TCGTTCGACCTGCGTAAAGT-3’
Cidea	5’-CTCGGCTGTCTCAATGTCAA-3’	5’-GGAACTGTCCCGTCATCTGT-3’

Supplementary Table S2. Within-group pre–post comparison of metabolic and performance parameters.

Group	Parameter	n	Pre (mean)	Post (mean)	Δ (Post–Pre)	95% CI lower	95% CI upper	Statistical test	p_value	Normality_p
CON	Grip Strength	8	128.375	147.625	19.25	10.06191653	28.43808347	Paired t-test	0.002	0.628
CON	Relative GS	8	503.75	508.125	4.375	-28.09306329	36.84306329	Paired t-test	0.759	0.284
CON	Exhaustive swimming time	8	5.46125	7.22625	1.765	-0.81402437	4.34402437	Paired t-test	0.150	0.633
CON	TCHO	8	84.875	86.75	3			Wilcoxon matched pairs	0.109	0.005
CON	LDL	8	16.5375	16.525	-0.0125	-3.109628295	3.084628295	Paired t-test	0.993	0.063
CON	TG	8	79.875	80.125	0.25	-0.615363867	1.115363867	Paired t-test	0.516	0.408
CON	Fasting glucose	8	149.375	139.875	-9.5	-46.80511267	27.80511267	Paired t-test	0.566	0.374
CON	OGTT AUC	8	25294.6875	25451.25	156.5625	-2295.794813	2608.919813	Paired t-test	0.884	0.366
HFD	Grip Strength	8	115.75	130.375	14.625	-4.465052442	33.71505244	Paired t-test	0.113	0.366
HFD	Relative GS	8	321.125	268.125	-53	-100.6563354	-5.343664635	Paired t-test	0.034	0.416
HFD	Exhaustive	8	6.015	8.92375	2.345			Wilcoxon	0.008	0.002

	swimming time							matched pairs		
HFD	TCHO	8	161.125	218	56.875	48.6661977	65.0838023	Paired t-test	<0.001	0.890
HFD	LDL	8	49.55	71.1625	21.6125	14.51758048	28.70741952	Paired t-test	<0.001	0.663
HFD	TG	8	95.375	115.125	19.75	17.71440126	21.78559874	Paired t-test	<0.001	0.279
HFD	Fasting glucose	8	200.75	178	-22.75	-59.65348245	14.15348245	Paired t-test	0.188	0.171
HFD	OGTT AUC	8	33911.25	35101.875	1190.625	-4655.154925	7036.404925	Paired t-test	0.645	0.535
15℃	Grip Strength	8	115.625	122.5	6.875	-9.196479097	22.9464791	Paired t-test	0.345	0.972
15℃	Relative GS	8	321.875	265.5	-56.375	-93.89877786	-18.85122214	Paired t-test	0.009	0.998
15℃	Exhaustive swimming time	8	6.425	31.27	20.795			Wilcoxon matched pairs	0.008	0.038
15℃	TCHO	8	162	198.125	36.125	27.68825703	44.56174297	Paired t-test	<0.001	0.363
15℃	LDL	8	49.475	64.2125	14.7375	8.41415293	21.06084707	Paired t-test	0.001	0.536
15℃	TG	8	95.875	106.875	11	7.425024164	14.57497584	Paired t-test	<0.001	0.783
15℃	Fasting glucose	8	191.75	191.25	-0.5	-40.59664514	39.59664514	Paired t-test	0.977	0.304
15℃	OGTT AUC	8	34139.0625	35321.25	1182.1875	-4947.470048	7311.845048	Paired t-test	0.662	0.101
25℃	Grip Strength	8	115.875	134.75	18.875	8.709890209	29.04010979	Paired t-test	0.003	0.262

25℃	Relative GS	8	326	320.75	-5.25	-38.75571484	28.25571484	Paired t-test	0.722	0.429
25℃	Exhaustive swimming time	8	6.08	14.18375	8.10375	5.852078594	10.35542141	Paired t-test	<0.001	0.430
25℃	TCHO	8	163.625	190.25	26.625	19.77549329	33.47450671	Paired t-test	<0.001	0.463
25℃	LDL	8	49.9625	55.0625	5.1	-4.578776674	14.77877667	Paired t-test	0.253	0.132
25℃	TG	8	96	102.75	6.75	3.86257067	9.63742933	Paired t-test	0.001	0.206
25℃	Fasting glucose	8	201	151.375	-49.625	-86.89781336	-12.35218664	Paired t-test	0.016	0.229
25℃	OGTT AUC	8	33928.125	36606.5625	2678.4375	-1456.628523	6813.503523	Paired t-test	0.169	0.828
32℃	Grip Strength	8	116.625	142	25.375	9.462733382	41.28726662	Paired t-test	0.007	0.343
32℃	Relative GS	8	322.375	332.5	10.125	-20.74941547	40.99941547	Paired t-test	0.463	0.865
32℃	Exhaustive swimming time	8	6.2475	14.36375	8.11625	6.578322456	9.654177544	Paired t-test	<0.001	0.234
32℃	TCHO	8	162.375	188.625	26.25	22.62647717	29.87352283	Paired t-test	<0.001	0.052
32℃	LDL	8	49.6	57.2	7.6	-4.059848224	19.25984822	Paired t-test	0.167	0.241
32℃	TG	8	95.25	100.625	5.375	2.733631244	8.016368756	Paired t-test	0.002	0.282
32℃	Fasting glucose	8	188.875	187.375	-1.5	-30.6530457	27.6530457	Paired t-test	0.907	0.386

32℃	OGTT AUC	8	34197.1875	31340.625	-2856.5625	-6100.201113	387.0761134	Paired t-test	0.076	0.775	

Data are presented as mean ± SD. Paired t-test or Wilcoxon matched-pairs test was applied depending on the normality of differences. $p<0.05$ was considered statistically significant.

TCHO = Total Cholesterol; LDL = Low-Density Lipoprotein Cholesterol; TG = Triglycerides; OGTT = Oral Glucose Tolerance Test; AUC = Area Under the Curve; GS = Grip Strength
